# Recycling micro polypropylene in modified hot asphalt mixture

**DOI:** 10.1038/s41598-023-30857-9

**Published:** 2023-03-04

**Authors:** Daniela Laura Buruiana, Puiu Lucian Georgescu, Gabriel Bogdan Carp, Viorica Ghisman

**Affiliations:** 1 Faculty of Engineering, Interdisciplinary Research Centre in the Field of Eco-Nano Technology and Advance Materials CC-ITI, “Dunarea de Jos” University of Galati, 47 Domneasca, 800008 Galati, Romania; 2 Faculty of Sciences and Environment, European Center of Excellence for the Environment, University of Galati, 800001 Galati, Romania

**Keywords:** Environmental sciences, Engineering

## Abstract

One of the objectives of the circular economy is solving the world’s plastic pollution crisis and recycling of materials by ensuring less waste. The motivation of this study was to demonstrate the possibility of recycling two types of wastes with a high risk of pollution, such as plastic based polypropylene and abrasive blasting grit wastes in asphalt roads. The effects of adding together polypropylene based microplastics and grit waste in asphalt mixture for wear layer performance have been shown in this study. The morphology and elemental composition of the hot asphalt mixture samples before and after freeze–thaw cycle were examined by SEM–EDX and the performance of the modified asphalt mixture was determined with laboratory tests including Marshall stability, flow rate, solid–liquid report, apparent density, and water absorption. A hot asphalt mixture suitable for making wear layer in road construction, containing aggregates, filler, bitumen, abrasive blasting grit waste and polypropylene based microplastics is also disclosed. In the recipe of modified hot asphalt mixtures were added 3 proportions of polypropylene-based microplastics such as 0.1%, 0.3% and 0.6%. An improvement of the mixture performance is shown at the asphalt mixture sample with 0.3% of polypropylene. In addition, polypropylene-based microplastics are bond with aggregates from mixture well, so the polypropylene-modified hot asphalt mixture can effectively decrease the appearance of cracks during sudden temperature changes.

## Introduction

Roads are the predominant transport infrastructure in Europe and an important contributor to the economy^1^. Hot-prepared asphalt mixture is a construction material made by a technological process that involves heating natural aggregates and bitumen, blending the mixture, transport, and commissioning, by hot compaction^2,3^. With the continuing accelerate climate change, various road degradation mechanisms including rutting, cracking, and erosion for sealed or unsealed roads were observed^4^. Asphalt or bituminous road mixtures generally use pre-washed river ballast as natural aggregates, mixed with quartz sands extracted from gravel pits or beds and crushed stone (taken from quarries because of rock crushing and sorting processes, or crushing sands fine fraction of career quarrels^5^. One of the Goal of 2030 AGENDA aims Sustainable Development to ensure sustainable consumption and production patterns. In order to support the concept of sustainable development in terms of restructuring the way natural resources are used, so that the economic activities balance with ecological systems, in order to avoid the latter total exhaustion in previous research we studied the usage of waste grit in asphalt mixture^6^. Shipyards use a material called grit with a composition of SiO_2_, Fe_2_O_3_, Al_2_O_3_, CaO, MgO, ZnO, MnO, SO_4_^–2^ and Cl in ship hull blasting operations. The waste grit resulting from the blasting process presents a major problem in terms of storage and environmental protection measures because the light fractions form flying dust (significant quantities are found transported on the banks of the Danube)^7^. In other study, we successfully replaced 25% of the amount of natural quartz sand with waste grit with similar granulometry (0.1–2.0 mm) and the asphalt mixture obtained presents both the physical–mechanical characteristics and the wear resistance superior of the standard asphalt mixture^8^. Plastic pollution is a serious issue of global concern that needs an urgent and international response involving all relevant members at various levels. Many studies are available on the use of recycled plastics in asphalt binders and mixtures^9^. Ahmed et al. evaluated the Low-Density Polyethylene (LDPE) and High-Density Polyethylene (HDPE) for asphalt modification via the wet process. They recommended the optimum dosage of 2% by weight of LDPE and HDPE in asphalt binder (40/50 penetration grade) for asphalt modification and the improvement of mixture properties was more marked for HDPE than LDPE^10^. Appiah et al. evaluated the use of recycled High-Density Polyethylene (HDPE) and polypropylene (PP) for asphalt modification through wet process. The optimum dosage identified was 2% HDPE and 3% PP by weight of asphalt binder (AC-20 grade). They obtained an increase of softening point and of viscosity of the base binder by adding HDPE and PP^11^. The second productive plastic is polypropylene defining for 21% of the total plastic market at the global level^12^. Compared to the polyethylene modifier, polypropylene is more challenging to mix homogeneously with asphalt through the wet process due to the higher melting point. The regular mixing temperature varies between 160 and 190 °C, while the percentage of polypropylene for modified asphalt production ranges among 3% and 5%^13^. Ahmedzade et al. have synthesised waste polypropylene additive which have a content of 80% wasted polypropylene and other materials (maleic anhydride and styrene) as modifier in bituminous binder amount of wt%: 3, 4, 5, and 6 by total weight of the binder, and they observed that at higher polymer content, mechanical behavior of binders are controlled by both bitumen and polymer phases which could induce an enhanced deformation behavior of bitumen under load^14^. Al-Hadidy and Yi-Giu investigated the benefits of modifying the asphalt and stone matrix asphalt (SMA) mixture in flexible pavement and they concluded that the pavement consisting of PP content of 5% by weight of asphalt is recommended for the improvement of the performance of asphalt concrete mixtures and PP-modified SMA as a surface layer is beneficial in reducing the construction materials^15^. The incorporation of ground polypropylene powders into asphalt would notably reduce the penetration and increase the viscosity and softening point of the modified binder blends, which has a pronounced rutting resistance than polyethylene modified asphalt^16^. Jin et al. conducted a study on using stamp sand and ASA plastic composite as pavement or road surfacing mixture. Their results showed that the stamp sand and acrylonitrile styrene acrylate waste mixtures have better moisture susceptibility and rutting resistance than the standard asphalt mixtures^17^. Solving the world's plastic pollution crisis is one of the objectives of circular economy. The concept of the circular economy presents the model supported by the production-consumption relationship involving the selection, reuse, revalorization, and recycling of materials and one of the aims consist in ensuring less waste. The potential asphalt products using plastic and grit wastes in order to demonstrate the decrease the economic and environmental costs, but also the CO_2_ emissions (Global Warming Potential-GWP in kg CO_2_ equivalent). Moreover, the costs production of the asphalt mixture is given by the prices of extraction of raw material, but also of the high price of the bitumen. Particularly, in S-E of Romania, sudden climate change with freeze–thaw cycle it causes damage to the asphalt, which leads to cracking. In order to overcome the above-mentioned issues, this study proposed the use of recycled polypropylene and waste grit in hot asphalt mixture. The experiment asphalt mixture samples explore the morphological characterization and the Marshall characteristics, such as stability, flow rate, solid–liquid report, apparent density, and water absorption. The novelty of our research consists in the mixture of both wastes, like polypropylene and grit from blasting process in hot asphalt mixture used as wear layer in road construction.

## Results and discussion

### Structural characterization of asphalt mixture samples

**Figure 1:**
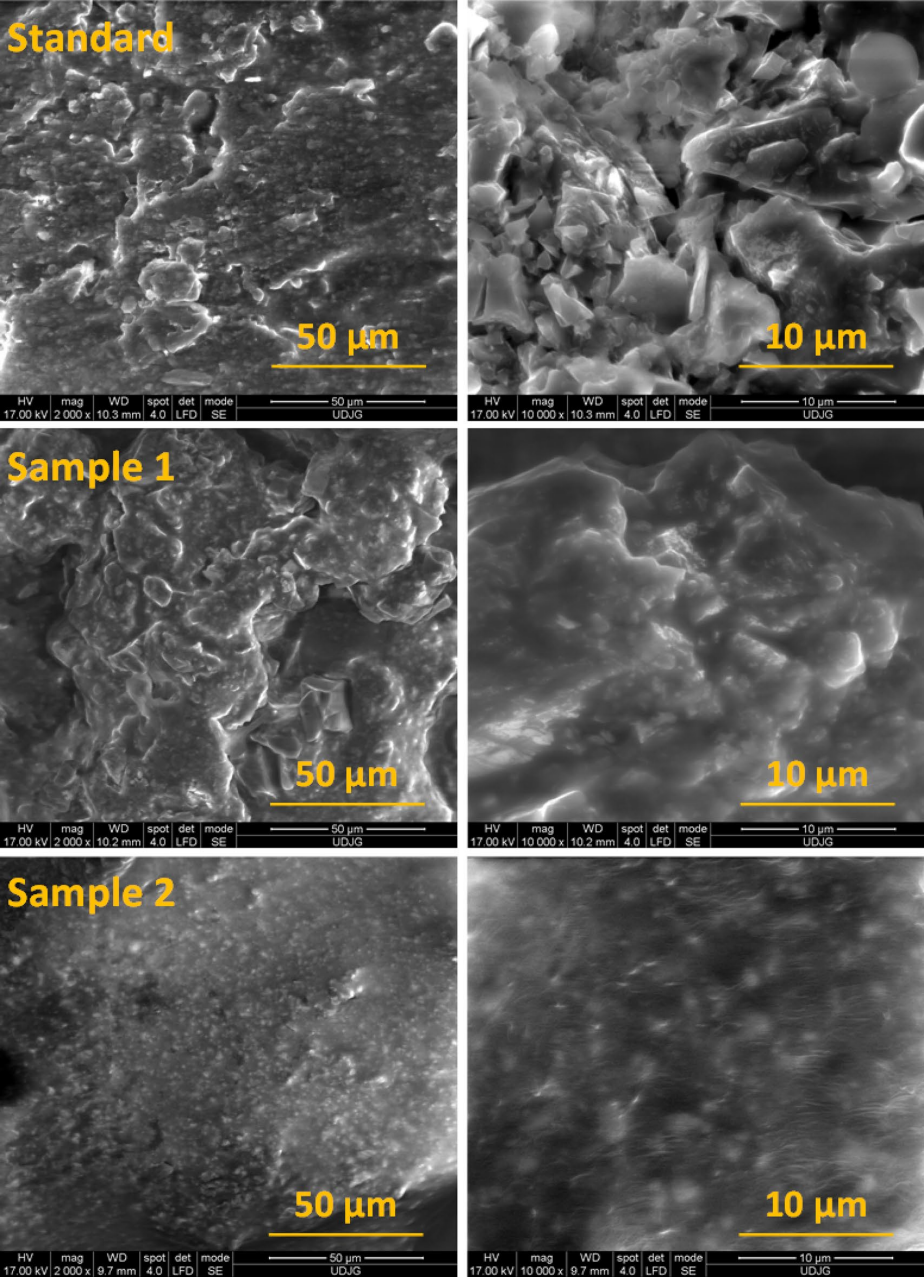
SEM images of standard, Sample 1 (0.1% PP) and Sample 2 (0.3% PP) hot asphalt mixture.

Figure 1 shows SEM images on the top of the Standard, Sample 1 and Sample 2 of hot asphalt mixture type BA8. As can be observed, the compact arrangement between the components when polypropylene (PP) is added (Sample 1 and Sample 2).

At standard sample can note a higher porosity compared to that of samples with PP. The morphology of the Sample 1 and Sample 2 shows that the crushed siliceous stone chipping, crushed sand, waste grit from blasting process and sort limestone filler are well embedded into a polymer matrix.

The embedding matrix and the bridges between the components show less pores and have a lower roughness.

**Figure 2:**
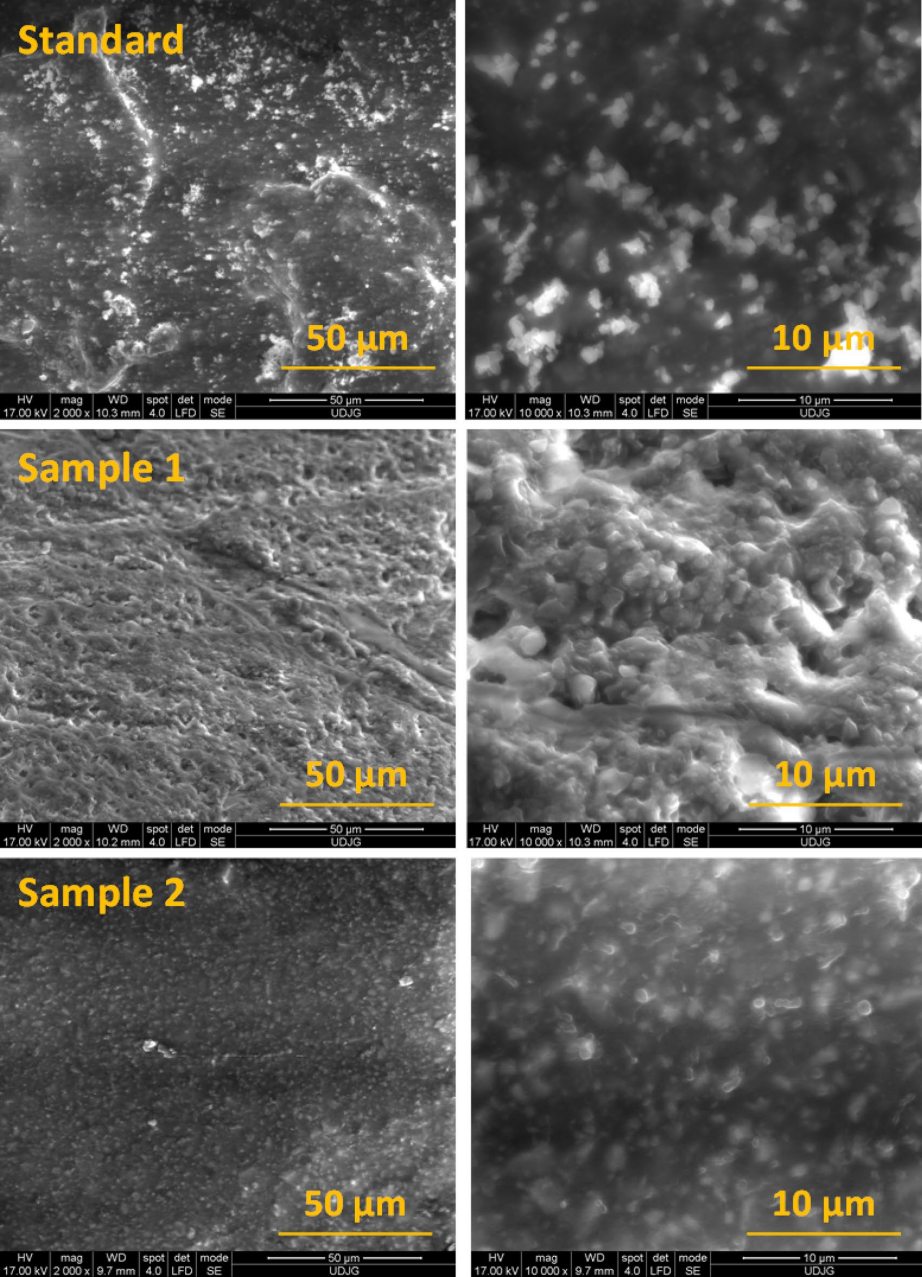
SEM images of standard, Sample 1 (0.1% PP) and Sample 2 (0.3% PP) hot asphalt mixture after freeze–thaw cycle.

SEM images on the top of the Standard, Sample 1 and Sample 2 of hot asphalt mixture type BA8 after freeze–thaw cycle are shown in Fig. 2. One can notice that in the case of Sample 2 (with higher% of polypropylene) the embedding polymer matrix is unchanged after freeze–thaw cycle, and we can say that present higher resistance to water induced damage at sudden climate change.

**Figure 3:**
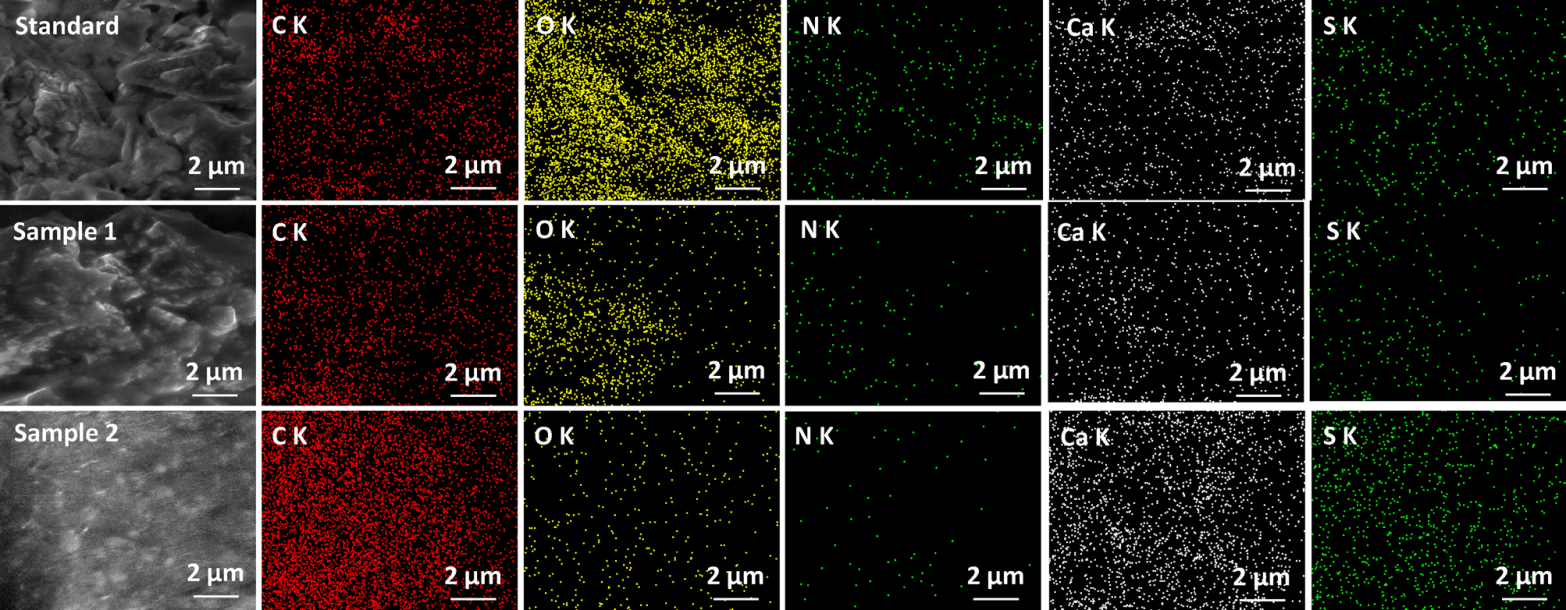
EDX elemental map of standard, Sample 1 (0.1% PP) and Sample 2 (0.3% PP) hot asphalt mixture.

The elemental composition consisting of C, O, N, Ca, and S is shown in Fig. 3. The high concentration of carbon in Sample 2 indicates the presence of polypropylene.

### Physical–mechanical characterization of asphalt mixture samples

#### Stability

Marshall stability measures the maximum load sustained by the mixture at a temperature of 60 °C and correlates well with in-service asphalt mixture rutting measurements.

**Figure 4:**
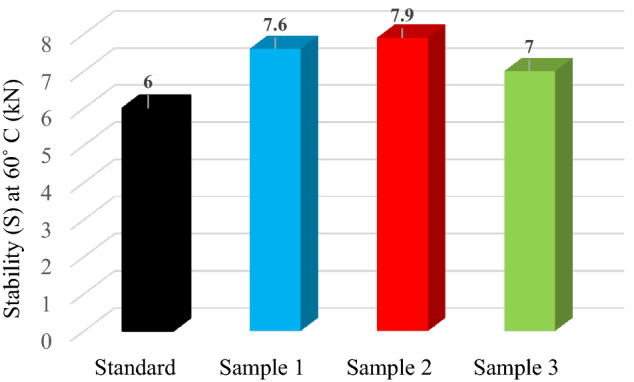
Stability at 60 °C for standard, Sample 1 (0.1% PP), Sample 2 (0.3% PP) and Sample 3 (0.6% PP) hot asphalt mixture.

According to STAS 174–197, for asphalt mixture type BA8 it is recommended that the Marshall stability is at least 6.0 kN. All the hot asphalt mixture testing results meet the performance requirement in Romania (Fig. 4). It shows that the rutting resistance increases first and then decreases with the increase of polypropylene content. As polypropylene content increases to 0.6% (Sample 3), the variance of gradation and mechanical properties of PP lowers the strength of the mixture.

The figure indicates that as PP content increases the stability increases and at higher content at 0.6% PP the Marshall stability decrease. This was attributed to the specific gravity of PP which is less than that of standard asphalt. This serves to penetrate between particles and enhanced interlock of aggregates, which increase stability. In the case of Sample 3 (0.6% PP) the stability decreases and can be related to the decrease in interlocking offered by microplastic PP-asphalt binder and aggregate particles. As a result, the mixture made with 0.3% PP (Sample 2) present the best value of the stability which leads to increasing resistance to deformation of the mixture.

#### Flow rate

**Figure 5:**
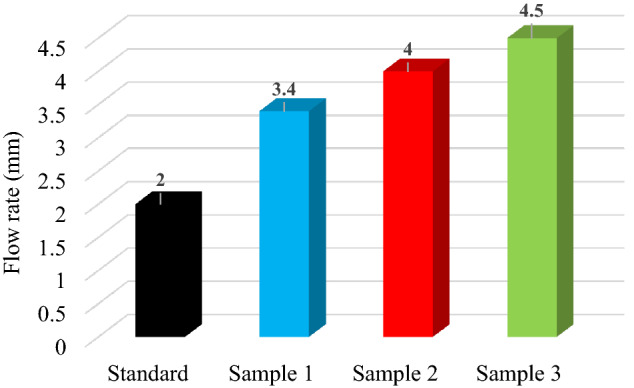
Flow rate for standard, Sample 1 (0.1% PP), Sample 2 (0.3% PP) and Sample 3 (0.6% PP) hot asphalt mixture.

The flow rate may vary from 1.5 to 4.5 mm, according to STAS 174–197. In Fig. 5 is shown the flow rate of standard and modified hot asphalt mixture samples. Figure 5 indicate that as PP content increases the flow rate increases. This was related to the decrease in interlocking offered by polypropylene-based microplastics in asphalt binder and aggregate particles.

Flow values increase with PP content indicating higher deformations under same pressure. This shows as polypropylene-based microplastics content increases, the resistance to deformation is lowered, particularly when PP content reaches 0.6%.

The flow values of asphalt mixture samples are higher than standard asphalt mixture, indicating higher deformations under the same pressure. Thus, the resistance to deformation of the asphalt mixture is reduced.

#### Solid–liquid report

**Figure 6:**
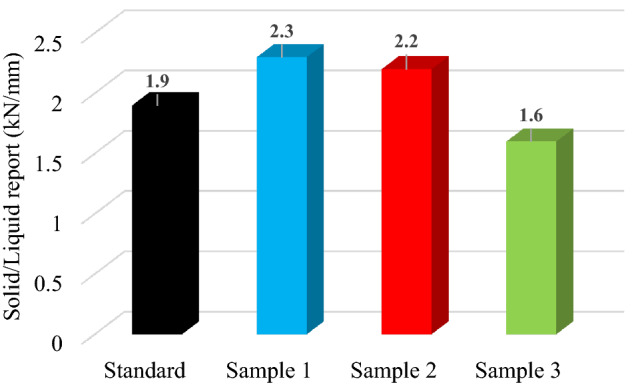
Solid–liquid report for standard, Sample 1 (0.1% PP), Sample 2 (0.3% PP) and Sample 3 (0.6% PP) hot asphalt mixture.

The minimum value for solid–liquid report required is 1.3 kN/mm. Figure 6 shows the solid–liquid report for standard and modified hot asphalt mixture samples.

All the asphalt mixture samples meet the performance requirement in Romania. The values for solid–liquid report decrease with increasing the content of polypropylene-based microplastics.

#### Apparent density

**Figure 7:**
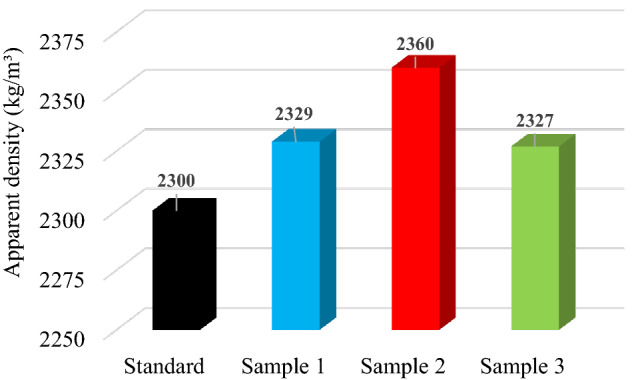
Apparent density for standard, Sample 1 (0.1% PP), Sample 2 (0.3% PP) and Sample 3 (0.6% PP) hot asphalt mixture.

In Fig. 7 is presented the apparent density for obtained asphalt mixture samples. For asphalt mixture type BA8 the value of apparent density should have at least 2.330 g/cm^3^ according to SR 174-1/2009. Performance of a wear layer is directly related to the density of the asphalt mixture. As can be seen the apparent density increases first and then decreases when the content of polypropylene leads to 0.6%. If the apparent density of the asphalt mixture is too low, rutting could develop as a result of low air voids due to wear layer densification under traffic.

To summarize this part, the asphalt mixture with 0.3% polypropylene-based microplastics yielded the highest apparent density.

#### Water absorption

**Figure 8:**
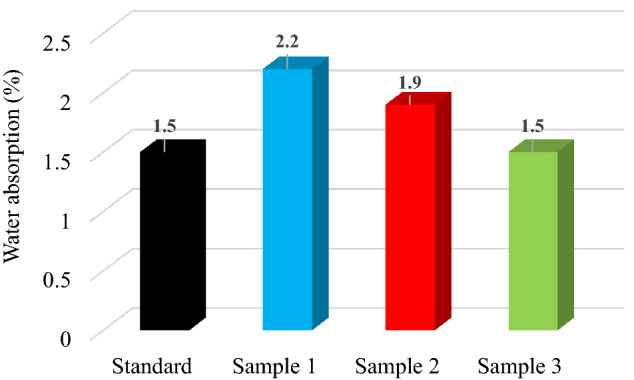
Water absorption for standard, Sample 1 (0.1% PP), Sample 2 (0.3% PP) and Sample 3 (0.6% PP) hot asphalt mixture.

Figure 8 shows the water absorption for standard and modified hot asphalt mixture samples.

According Romanian Standard STAS 174-1/2009, the value of water absorption should be ranged between 1.5 and 5.0%. In the case of sample with the small percentage of polypropylene-based microplastics water absorption is higher and decreases with the increase of polypropylene content.

## Materials and methods

### Sample preparation

The type of asphalt mixture used in this study was wear layers (rolling) called asphalt concrete with crushed stone BA 8, where 8 represent the maximum size of the granule^3^. The hot asphalt mixture samples type BA8 were made according to standard STAS 11348-87^18^ obtained in the road-testing laboratory within the Tancrad Company. The weight proportions of the components of standard asphalt mixture sample consists of 33.5% crushed siliceous stone chipping with a granulation ranging between 4.0 and 8.0 mm, 50% crushed sand with a granulation ranging between 0.1 and 4.0 mm, 10% sort limestone filler and 6.5% road bitumen type 50/70^18,19^. For Marshall test were prepared three recipes by replacing 25% crushed sand with a granulation ranging between 0.1 and 4.0 mm with 25% waste grit from blasting process with a granulation ranging between 0.1–2.00 mm and by adding weight percentage of polypropylene (PP)-based microplastics with granulation ranging between 0.1 and 2 mm. Thus, the weight proportions of the components of asphalt mixtures samples consists of 33.5% crushed siliceous stone chipping with a granulation ranging between 4.0 and 8.0 mm, 25% crushed sand with a granulation ranging between 0.1 and 4.0 mm, 25% waste grit from blasting process with a granulation ranging between 0.1 and 2.00 mm, 10% sort limestone filler and 6.4% road bitumen type 50/70 with 0.1% polypropylene-based microplastics for Sample 1; 6.2% road bitumen type 50/70 with 0.3% polypropylene-based microplastics for Sample 2 and 5.9% road bitumen type 50/70 with 0.6% polypropylene-based microplas-tics for Sample 3, as can be seen listed in Table 1. The recipe of Sample 2 was the subject of the patent in collaboration with economic environment^20^.

**Table 1:** Recipes for hot asphalt mixture samples type BA8.

Component	Standard	Sample 1	Sample 2	Sample 3
Crushed siliceous stone chipping, (%)	33.5	33.5	33.5	33.5
Crushed sand, (%)	50	25	25	25
Waste grit, (%)	–	25	25	25
Sort limestone filler, (%)	10	10	10	10
Road bitumen type 50/70, (%)	6.5	6.4	6.2	5.9
Polypropylene-based microplastics, (%)	–	0.1	0.3	0.6

The obtained samples have cylindrical form with a diameter of 10 cm and a height of 6.3 cm.

**Figure 9:**
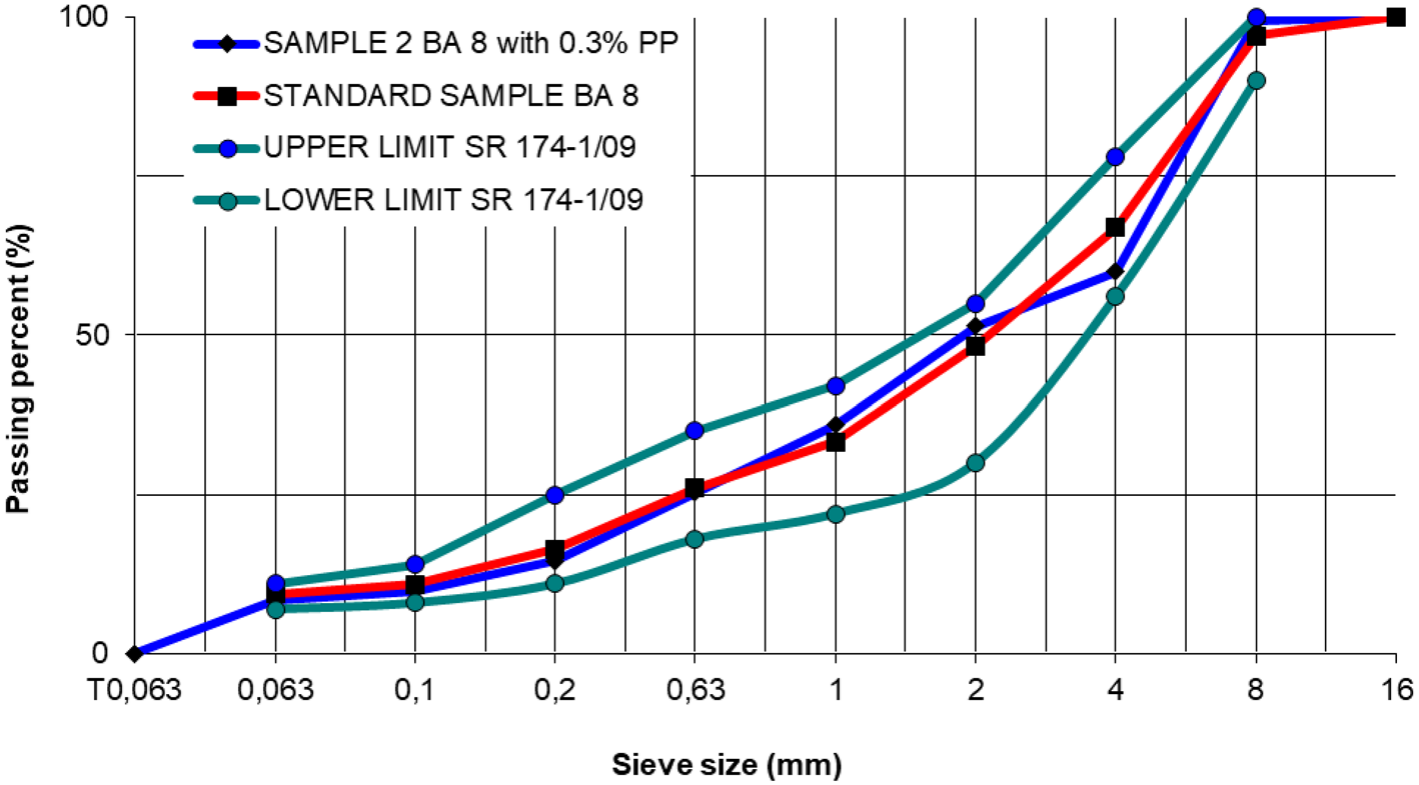
Mixture gradation of standard and Sample 2 (0.3% PP) hot asphalt mixture type BA 8.

The asphalt mixture BA 8 was used in this study, which is a typical asphalt concrete with crushed stone widely employed, its gradation is shown in Fig. 9. The gradation of the asphalt mixture was made for standard sample and for Sample 2 with 0.3% polypropylene-based microplastics. The content of the binder was determined by the Marshall method according to Romanian Standard SR 174-1/2009^21^.

### Characterization technique

The morphology and elemental composition of the hot asphalt mixture samples before and after freeze–thaw cycle were examined by scanning electron microscopy coupled with energy dispersive X-Ray (SEM/EDX) spectroscopy using a FEI Q 200 microscope in low vacuum. Before examination, the hot asphalt mixture samples were coated with 4 nm thick conducting layer of Au using a SPI-Module™ sputter coater system.

To verify the quality of the asphalt mixtures, the physical–mechanical characteristics were determined by tests on Marshall cylinders. The principle of the test consists in determining the breaking strength of a cylindrical specimen, under a force applied to a generator. The test is performed on the sample placed in the cast at a temperature of 60 °C.

The physical–mechanical characteristics were performed according to Romanian Standard SR 174-1/2009^21^ as follows:

- The stability (S) is the load, expressed in kN, reached at the moment when the cylindrical specimen breaks at a temperature of 60 °C.
- The flow index (I), is the deformation reached by the vertical diameter of the specimen at the time of breaking and is expressed in mm.
- The apparent density (g/cm^3^) represents the mass of the volume unit of the compacted asphalt mixture, which includes the voids filled with air. This is determined based on the sample mass/volume formula, according to the relationship (1):
  1 $${\rho }_{a}=\frac{{m}_{u}}{V} \left(\frac{\mathrm{g}}{{\mathrm{cm}}^{3}}\right),$$
  where, for the standard sample, we have: ${\rho }_{a}$ is the apparent density of the asphalt mixture, (g/cm^3^); m_u_ is the mass of the sample weighed on the analytical balance in a dry state and which has a value of 1137.4 g; V is the volume of the cylindrical sample with a diameter of 10 cm and a height of 6.3 cm is 494.5 cm^3^.

Water absorption (%) is the amount of water absorbed by the externally accessible voids of an asphalt mixture specimen, and was determined by evaporation after immersion in water, using the thermal method in static mode. Initially, after removing the sample from the water, it was wiped with a damp fabric to remove the excess surface and gravity water. The wet sample was placed in an oven, where it was dried at a temperature of 60 °C and RH below 2.5%. Water absorption or wetting capacity is expressed as a percentage of the initial mass of the sample and the wetted mass. Water absorption, in volume percentage, is calculated with the relation:

2 $$A=100\frac{{m}_{i}}{{m}_{f}},$$

where m_i_ is the initial mass of the sample; m_f_ is the final mass of the sample after it has been moistened.

In this study, every test result consisted of the average of three replicate tests.

## Conclusions

One of the solutions to solve urgent environmental issues regarding plastic pollution is promoting plastic waste recycling to reduce climate and environment impact. It was shown that the use of waste materials, like polypropylene and abrasive blasting grit in hot asphalt mixture is a viable option that needs to be implemented further. Test results indicated that adding together polypropylene-based microplastics and grit waste improved the mixture performance.

SEM analysis shows that in the case of the hot asphalt mixture with 0.3% PP the embedding polymer matrix is unchanged after freeze–thaw cycle, and we can say that present higher resistance to water induced damage at sudden climate change. The relationship between Marshall properties and microplastic based polypropylene content is reflected through that as PP content increases the stability increases which is related to penetration of PP between particles and enhanced interlock of aggregates. Based on the obtained Marshall characteristics 0.3% was selected as the optimum content for polypropylene.

In conclusion, the polypropylene-based microplastics behave like a homogeneous material which stabilize the particles of waste sandblasting grit and reduce the appearance of cracks in hot asphalt mixture after freeze–thaw cycles.

In future studies, we intend to experimentally pour a portion of road with the proposed asphalt mixture recipe and monitor it under climatic conditions to support this study.

## Supplementary Information

Supplementary Figures.

## Data Availability

All data analysed during this study are included in this published article and its Supplementary Information files.
