# Supplementary material for: Recycling micro polypropylene in modified hot asphalt mixture
Source: Sci Rep. 2023 Mar 4;13:3639. doi: 10.1038/s41598-023-30857-9 (PMC9985627; doi:10.1038/s41598-023-30857-9)
Supplement: Supplementary file 1 — Supplementary Figures. [file 41598_2023_30857_MOESM1_ESM.docx]

Recycling Micro Polypropylene and Sandblasting Grit in Hot Asphalt Mixture

Daniela Laura Buruiana^1^, Puiu Lucian Georgescu^2^, Gabriel Bogdan Carp^1^, Viorica Ghisman ^1,*^

^1^ Interdisciplinary Research Centre in the Field of Eco-Nano Technology and Advance mate-rials CC-ITI, Faculty of Engineering, “Dunarea de Jos” University of Galati, 47 Domneasca, 800008 Galati, Romania;

^2^ European Center of Excellence for the Environment, Faculty of Sciences and Environment, University of Galati, 800001 Galati, Romania;

*Correspondence to*: Viorica Ghisman ([viorica.ghisman@ugal.ro](mailto:Daniela.buruiana@ugal.ro))

The EDX spectra of the raw data of hot asphalt mixture samples are presented in Fig. S1-S3 recorded over the entire surface of the analysed area.

**Figure S1:** EDX spectra of hot asphalt mixture sample (Standard).

**Figure S2:** EDX spectra analysis of hot asphalt mixture (Sample 1).

**Figure S3:** EDX spectra analysis of hot asphalt mixture (Sample 2).
